# Delineating the Relationships Between Motor, Cognitive-Executive and Psychiatric Symptoms in Female *FMR1* Premutation Carriers

**DOI:** 10.3389/fpsyt.2021.742929

**Published:** 2021-12-03

**Authors:** Darren R. Hocking, Danuta Z. Loesch, Paige Stimpson, Flora Tassone, Anna Atkinson, Elsdon Storey

**Affiliations:** ^1^Developmental Neuromotor and Cognition Lab, School of Psychology and Public Health, La Trobe University, Melbourne, VIC, Australia; ^2^School of Psychology and Public Health, La Trobe University, Melbourne, VIC, Australia; ^3^Psychology Department, Monash Health, Clayton, VIC, Australia; ^4^Department of Biochemistry and Molecular Medicine, School of Medicine and M.I.N.D. Institute, University of California Davis Medical Center, University of California, Davis, Davis, CA, United States; ^5^Department of Medicine (Neuroscience), Monash University, Alfred Hospital Campus, Melbourne, VIC, Australia

**Keywords:** fragile X premutation, response inhibition, executive function, psychiatric symptoms, ataxia, cerebellum, motor, Fragile X-Associated Tremor/Ataxia Syndrome (FXTAS)

## Abstract

**Introduction:** Premutation expansions (55–200 CGG repeats) of the Fragile X Mental Retardation 1 (FMR1) gene on the X chromosome are associated with a range of clinical features. Apart from the most severe - Fragile X-Associated Tremor/Ataxia Syndrome (FXTAS) - where the most typical white matter changes affect cerebellar peduncles, more subtle changes may include impairment of executive functioning, affective disorders and/or subtle motor changes. Here we aimed to examine whether performance in selected components of executive functioning is associated with subclinical psychiatric symptoms in non-FXTAS, adult females carrying the *FMR1* premutation.

**Methods and Sample:** A total of 47 female premutation carriers (sub-symptomatic for FXTAS) of wide age range (26–77 years; M = 50.3; SD = 10.9) were assessed using standard neuropsychological tests, three motor rating scales and self-reported measures of psychiatric symptoms using the Symptom Checklist-90-Revised (SCL-90-R).

**Results:** After adjusting for age and educational level where appropriate, both non-verbal reasoning and response inhibition as assessed on the Stroop task (i.e., the ability to resolve cognitive interference) were associated with a range of primary psychiatric symptom dimensions, and response inhibition uniquely predicted some primary symptoms and global psychiatric features. Importantly, lower scores (worse performance) in response inhibition were also strongly correlated with higher (worse) scores on standard motor rating scales for tremor-ataxia and for parkinsonism.

**Conclusion:** These results provide evidence for the importance of response inhibition in the manifestation of psychiatric symptoms and subtle tremor-ataxia motor features, suggestive of the presence of early cerebellar changes in female premutation carriers.

## Introduction

The premutation expansion (PM: 55–200 CGG repeats) of the Fragile X Mental Retardation 1 (*FMR1*) gene on the X chromosome is associated with a range of clinical features, including executive function impairments and affective disorders (1). The most severe form of clinical disorder associated with premutation expansions in the 5′ untranslated region of the Fragile X Mental Retardation 1 (*FMR1*) gene is a late-onset progressive neurodegenerative condition: Fragile X-Associated Tremor/Ataxia Syndrome (2). The clinical features include intention and/or postural tremor, gait ataxia, dementia, and in some cases parkinsonism. This syndrome usually occurs after, and progresses from, the age of 55 years, and is more prevalent in male (45%) than in female (8–16%) PM carriers. In addition to FXTAS, women carrying the PM allele are at increased risk (~20%) of developing premature ovarian failure (3). Apart from these two well-recognized disorders, subtle cognitive-executive and neuropsychiatric features are not uncommon (4), and the latter have more recently been recognized as a distinct group termed: Fragile X-Associated Neuropsychiatric Disorders (FXAND) (5). These include cognitive-executive impairments, anxiety, social deficits, depression and obsessive compulsive features.

Although the presence of a cognitive-executive phenotype in female PM carriers has been controversial in the literature, deficits have been reported in the area of response inhibition, using direct behavioral assessment and oculomotor antisaccade paradigms (6–9). Response inhibition is an aspect of executive function that enables suppression of a prepotent response that is inappropriate in a given context (10). Several studies documented age-dependent changes in response inhibition deficits in female PM carriers, with older age associated with slower inhibition of prepotent verbal responses and longer antisaccade latency (6, 8). However, some of these findings reported in the literature have been inconsistent (11, 12), perhaps owing to selection bias, choice of response inhibition measure, discrepant age ranges, and small sample sizes.

Apart from cognitive-executive issues, the effect of the PM alleles has been documented in elevated prevalence of some psychiatric disorders, especially mood and anxiety disorders (4), with elevated symptoms of social phobia, obsessive-compulsive behavior, somatization and anxiety/depression, in female PM carriers with or without the environmental stressors arising from raising a child with Fragile X syndrome (4, 13, 14). With regard to trajectories in female PM carriers, there is evidence for increasing deterioration of psychiatric problems especially in anxiety and obsessive-compulsive symptoms (15), and increasing prevalence and severity of mood and anxiety disorders over time (4). Notably, poorer inhibition of verbal responses have been linked to self-reports of elevated anxiety and depression in non-FXTAS asymptomatic female PM carriers (7), suggesting that subtle cognitive-executive difficulties may contribute to or co-occur with psychiatric features.

Previous findings that impairments in a range of motor domains are associated with increasing CGG repeat size in otherwise unaffected PM carriers (16, 17) indicate that multiple sensorimotor issues correspond with increased disease risk prior to development of prominent symptoms and observable clinical signs. Several studies have reported subtle sensorimotor deficits in apparently asymptomatic (non-FXTAS) female PM carriers, including changes in upper limb manual control (18), step initiation (19), gait function, and postural control (20–22). Importantly, other studies have documented associations between gait, stepping/postural control and cognitive-executive functions in PM carriers with and without FXTAS (19–21).

Delineating the female PM carrier phenotype at multiple severities across motor, cognitive-executive, and psychiatric domains will be important for examination of synergistic effects of gender, aging and the *FMR1* premutation allele on these subclinical features.

In a recent study, we have shown that mild motor deficits (cerebellar ataxia and tremor scores) were correlated with set-shifting, working memory and psychomotor speed in female PM carriers without a diagnosis of FXTAS (23). Thus, there is converging evidence for the view that there is a continuum of subclinical changes that accumulate over the lifetime, irrespective of the final clinical endpoint. However, there has been no studies considering the inter-relationships between all three domains—motor, cognitive-executive and neuropsychiatric—in female PM carriers within the same study.

The present study explores the relationships between distinct subcomponents of cognitive-executive functioning and a range of psychiatric symptoms in a sample of non-FXTAS, clinically unaffected female PM carriers across a broad range of age and CGG expansion sizes. Based on previous literature, we tested the hypothesis that poorer inhibitory control will be correlated with higher levels of psychiatric symptoms in the absence of formal diagnoses. We have also considered a specific relationship between inhibitory control and subtle motor features of tremor-ataxia in female PM carriers. We hypothesized that worse motor rating scores will be correlated with reduced cognitive inhibition performance at a subclinical but detectable level in female PM carriers.

## Methods and Sample

### Participants

The study protocol was approved by the La Trobe University and Monash University Human Research Ethics Committees and informed consent was provided by all participants. The sample comprised a total of 47 adult female PM carriers aged between 26 and 77 years (mean = 50.3; SD = 10.9, CGG repeats from 56 to 133), recruited through cascade testing of a large cohort of fragile X families as described in our previous studies (24–26). All participants were white Caucasian with the exception of one participant of Asian descent. The sample characteristics of the female PM carriers is provided in Table 1. On the basis of FXTAS criteria, none of the female PM carriers met diagnostic criteria for inclusion as FXTAS. Based on formal testing by two neurologists (ES & DZL), elevated scores on standard neurological motor rating scales (see below) were identified in 27 female PM carriers; however, we classified this increase as “sub-symptomatic for FXTAS” based on standard testing using motor and cognitive scale scores as previously described (23).

**Table 1:** Demographic, motor and cognitive-psychiatric characteristics.

				**Normative values** ^**a**^	
**Variable**	**Mean**	**SD**	**Range**	**Mean**	**SD**
**Characteristic**					
Age	50.3	10.9	26–77		
Age of menopause	43.6	7.1	27–53		
Year of Education	12.9	3.3	4–22		
CGG repeats	82.7	16.5	56–133		
**Motor Score**					
UPDRS	2.5	3.3	0–17	1.9	2.0^b^
ICARS Total	6.7	3.6	2–16	4.1	2.2^c^
**Cognitive measures**					
Vocab SS	9.8	2.5	4–17	10.5	3.2^d^
MR SS	12.2	2.9	5–17	10.4	2.9^d^
DS Forwards	6.5	1.2	4–9	7.6	0.8^e^
DS Backwards	4.9	1.4	3–8	3.1	1.2^e^
Pro-rated IQ	103.6	13.2	74–132	–	–
TMT A (raw score)	35.8	13.3	17–105	31.8	9.9^f^
TMT B (raw score)	79.3	31.3	37–168	78.8	19.1^f^
TMT B-A	46.0	26.8	8–128	32.0	14.4^f^
Stroop (t-score)	51.9	9.2	34–71	43.9	23.1^g^
SDMT (raw score)	51.0	10.5	33–74	47.9	10.6^h^
**SCL-90-R Scales (t scores)** ^**i**^					
Somatization	49.3	10.1	35–79	46.6	8.4
Depression	50.0	9.5	34–69	49.0	11.2
Interpersonal sensitivity	50.7	9.3	39–71	50.5	10.4
Phobic anxiety	47.2	6.7	41–66	46.9	8.3
Paranoid ideation	48.3	8.6	41–65	50.0	8.6
Obsessive-compulsive	51.7	10.0	37–74	51.7	9.3
Hostility	47.3	6.8	35–65	47.1	7.5
Anxiety	46.3	8.9	37–65	46.0	8.6
**Global Severity Index (GSI)**	48.7	11.3	30–69	–	–

*^a^Normative data derived from relevant studies where available.*

*^b^Postuma et al. (27).*

*^c^Fitzpatrick et al. (28).*

*^d^Harrison et al. (29).*

*^e^Leung et al. (30).*

*^f^Tombaugh (31).*

*^g^Bayard et al. (32).*

*^h^Kiely et al. (33).*

*^i^Gossett et al. (14).*

*Vocab SS, WAIS-III Vocabulary Scaled Score; MR SS, WAIS-III Matrix Reasoning Scaled Score; DS Forwards, WAIS-III forwards digit span subtotal; DS Backwards, WAIS-III backwards digit span subtotal; TMT-A and -B, Trail-Making Test A and B; SDMT, Symbol-Digit Modalities Test*.

### Cognitive-Executive Measures

The cognitive measures were selected to focus on domains that relied on executive functioning, non-verbal reasoning and psychomotor speed, which are domains of impairment observed in male and female PM carriers [see (1) for a review]. General cognitive ability was assessed by the Vocabulary and Matrix Reasoning subtests of the Wechsler Adult Intelligence Scale (Third Edition; WAIS-III), which were used to calculate a prorated Full Scale IQ score (34). We used the Matrix Reasoning subtest as a measure of non-verbal reasoning. The WAIS-III Digit Span forward and backward components, assessed separately, were selected as measures of short-term verbal memory and working memory, respectively. Task or set switching was measured using the Trail Making Test (35). The Stroop Color-Word Test (36) was used as a measure of response inhibition in which the participant is asked to name the (ink-printed) color of a series of words of different colors (blue, green, red, brown, purple) while selectively inhibiting the automatic response of reading the names of printed color words aloud. For example, the word “blue” might be printed in green ink with the participant instructed to respond “green.” Time to complete the task (typically <10 min for the Victoria version) was recorded and a decrease in color-naming speed was taken as measure of increased color-word interference. Finally, the Symbol Digit Modalities Test was used as a measure of psychomotor speed (37).

### Motor Rating Scales

Tremor-ataxia motor signs were identified and scored using the International Cooperative Ataxia Rating Scale (ICARS) (38); and parkinsonian features using the Unified Parkinson's Disease Rating Scale (UPDRS) Part III-Motor (39). These standard neurological motor rating scales, with established inter-rater reliability (40–42) were administered and scored by two neurologists (ES & DZL) with relevant experience in administering these scales from previous studies.

### Psychiatric Symptom Measures

The SCL-90-R is a 90 item self-report questionnaire measuring a range of symptom clusters and general psychiatric symptomatology occurring over the past week (43). The 90 items are clustered into nine primary symptom dimensions: somatization, obsessive compulsive, interpersonal sensitivity, depression, anxiety, hostility, phobic anxiety, paranoid ideation, and psychoticism. The Global Severity Index (GSI) is an overall measure of the level of psychological distress and intensity of symptoms, and is calculated from the average of the primary symptom scales (43). It has good internal consistency with coefficient alphas ranging from 0.77 to 0.90 across scales, and test-retest reliability over a period of 1 week from 0.80 to 0.90. We report T-scores for both the symptom dimension scales and the overall level of psychiatric disturbance (GSI T-score), with a score between 60 and 63 considered borderline and above 63 classified as above clinically significant threshold.

### Genetic Molecular Measures

#### CGG Repeat Size

Standard methods were employed to isolate genomic DNA from peripheral blood lymphocytes using polymerase chain reaction and Southern blot analyses as described in our previous publications (23). Briefly, the Southern blot analysis involved digestion with 10 micrograms (μg) of isolated DNA using EcoRI and NruI. The *FMR1* genomic dig-labeled StB12.3 probe was used for hybridization as previously described (44). PCR was used to amplify the genomic DNA (45).

### Statistical Analysis

Data were analyzed using the Statistical Package for the Social Sciences version 26 (SPSS; IBM Corporation; Armonk, NY, USA). First, outliers were identified using boxplots, histograms, and by examining Z-scores. There were no extreme outliers removed from the dataset and standardized values were within the ranges −3.29 to 3.29. The distributions of scores for the motor measures and the cognitive measures were then evaluated for deviations from normality using Shapiro-Wilk's test of normality, disclosing some violations of the assumption of normality as required for parametric tests. Second, we conducted Spearman's rank correlation (ρ) to examine correlations between motor scores, cognitive-executive measures and psychiatric symptom scores, after adjusting for age and/or year of education (where appropriate). We then used the Holm-Bonferroni family-wise false discovery rate (FWFDR) correction method to correct the *p*-values for multiple comparisons.

The final approach consisted of a series of linear multiple regressions which were applied to identify the most significant predictors of psychiatric symptomatology on the SCL-90-R. This analysis was based on selected cognitive-executive variables found to be significantly related to psychiatric scores in correlational analysis. Age and years of education were entered as independent control variables. The assumptions of singularity and multicollinearity were met (*r* <0.7), and inspection of residual scatterplots indicated assumptions of normality, linearity, homoscedasticity, and independence of residuals were also met. Cook's distances were less than one and values of the standardized residuals were between −3 and 3.

## Results

### Sample Characteristics

Table 1 shows the sample characteristics for the cognitive-executive and SCL-90-R dimension scales and Global Severity Index. There was a single participant aged 26 years; the majority of the participants were aged between 35 and 50 years. CGG repeat length ranged from 56 to 133. We reported in a previous study (23) that the scores on the ICARS and the UPDRS were elevated in a proportion of the female carriers from the same sample as in the current study. However, we categorize this increase as sub-symptomatic for FXTAS since it had not generated any specific medical diagnoses, or realization of abnormality on the part of those individuals presenting with evidently abnormal scores.

As seen in Table 1, the scores on the cognitive measures were mostly comparable to normative values taken from relevant studies, with the exception of higher Stroop Color-Word Test t-scores in female PM carriers. On the SCL-90-R Global Severity Index, there were six participants who scored above the clinically significant threshold of 63, and another five participants scored in the borderline clinical range. However, the mean scores for premutation females on dimension scales of the SCL-90-R were comparable to control values as reported in Gossett et al. (14).

### Correlations Between Motor, Cognitive and Psychiatric Symptom Scores

**Figure 1:**
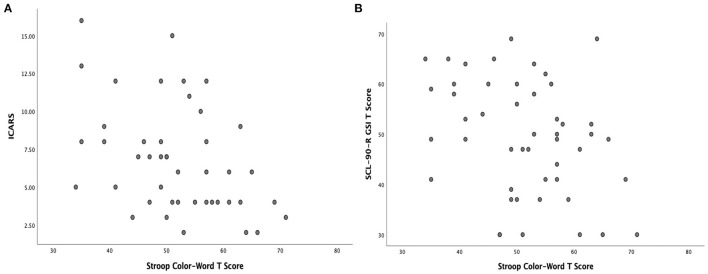
**(A)** Scatterplots illustrating significant relationship between Stroop Color-Word t-score and ICARS Total score and **(B)** Scatterplots illustrating a significant relationship between Stroop Color-Word t-score and SCL-90-R Global Severity Index (GSI) t-score.

Spearman's rank correlations between cognitive-executive and SCL-90-R scores are provided in Table 2. There were significant negative correlations between Matrix Reasoning scaled scores (higher is better) and the Hostility symptom scale (higher is worse) (*p* = 0.003). Stroop Color-Word Test t-scores were significantly negatively correlated with scores on several of the SCL-90-R dimension scales (Depression, Hostility, Anxiety), and with the Global Severity Index (between *p* = 0.019–0.046). Considering the multitude of relationships of the Stroop Color-Word test with psychiatric symptom scales, we additionally explored the relationship of these scores with the motor scale scores and found that Stroop t-scores were significantly (negatively) correlated with both ICARS and UPDRS (ρ = −0.50, *p* = 0.001, with ICARS; ρ = −0.44, *p* = 0.003, with UPDRS). The scatterplots illustrating the nature of these relationships are shown in Figure 1.

**Table 2:** Spearman's rank correlation (ρ) between cognitive-executive measures and SCL-90-R psychiatric symptoms, adjusted for age and/or year of education (whenever appropriate).

	**MR SS** ^**#**^		**DS forwards** ^**#**^		**DS backwards**		**Stroop**		**TMT B-A** ^**γ**^		**SDMT** ^**γ**^	
	**ρ**	***p-*value**	**ρ**	***p-*value**	**ρ**	***p-*value**	**ρ**	***p-*value**	**ρ**	***p-*value**	**ρ**	***p-*value**
Somatization	−0.06	0.715	0.00	0.999	−0.10	0.500	−0.29	0.050	−0.24	0.125	−0.02	0.895
Depression	−0.25	0.093	0.08	0.594	0.08	0.604	−0.37	0.036*	−0.04	0.811	−0.09	0.582
Interpersonal	−0.22	0.155	−0.03	0.848	0.05	0.728	−0.20	0.183	−0.12	0.473	−0.06	0.723
Phobic anxiety	−0.05	0.723	−0.08	0.611	−0.18	0.241	−0.23	0.129	−0.01	0.998	−0.07	0.671
Paranoid ideation	−0.19	0.210	0.04	0.817	−0.06	0.690	−0.19	0.220	−0.08	0.629	−0.02	0.878
Obsessive-compulsive	−0.20	0.195	0.16	0.306	−0.04	0.786	−0.19	221	−0.14	0.420	−0.01	0.997
Hostility	−0.43	0.003*	0.10	0.504	−0.21	0.159	−0.30	0.046*	−0.14	0.396	−0.02	0.916
Anxiety	−0.20	0.179	0.01	0.970	−0.23	0.135	−0.31	0.041*	−0.10	0.521	−0.05	0.768
Psychoticism	−0.19	0.222	0.09	0.540	0.01	0.939	−0.16	0.286	−0.05	0.744	−0.01	0.971
Global Severity Index	−0.28	0.065	0.02	0.973	0.03	0.831	−0.34	0.019*	−0.15	0.362	−0.06	0.727

*p-values remain α = 0.05 after adjustment using the Holm-Bonferroni family-wise false discovery rate (FWFDR) correction method. Bolded p-values indicate that correlation is significant after FWFDR correction.

*^#^corrected for years of education.*

*^γ^corrected for age and years of education.*

*Note there were significant correlations between Stroop Color-Word T Score and both ICARS (ρ = −0.503, p = 0.001) and UPDRS (ρ = −0.436, p = 0.003), after adjusting for age.*

*MR SS, WAIS-III Matrix Reasoning Scaled Score; DS Forwards, WAIS-III forwards digit span subtotal; DS Backwards, WAIS-III backwards digit span subtotal; Stroop, Stroop Color-Word T Score; TMT B-A, Trail-Making Test time difference between B and A; SDMT, Symbol-Digit Modalities Test*.

### Multiple Linear Regression Analysis for Cognitive-Executive Variables Predicting Psychiatric Symptom Scores

The multiple regression analysis showed that the Stroop Color-Word Test scores were a significant predictor of primary psychiatric symptom dimensions, Somatization (β = −0.35, *p* = 0.049) and Phobic Anxiety (β = −0.34, *p* = 0.049) (Table 3), and overall severity of psychiatric symptoms (β = −0.33, *p* = 0.017) on the SCL-90-R Global Severity Index (Table 4).

**Table 3:** Multiple linear regression analyses for cognitive-executive variables predicting SCL-90-R symptom scale scores in female premutation carriers.

	**Somatization**				**Phobic anxiety**			
**Predictor**	***B***	***SE B***	**β**	***t***	***B***	***SE B***	**β**	***t***
Age	−0.149	0.150	−0.164	−0.992	−0.082	0.094	−0.144	−0.875
YOE^a^	−0.723	0.587	−0.218	−1.230	−0.581	0.367	−0.279	−1.582
MR SS^b^	0.584	0.688	0.158	0.849	0.755	0.430	0.325	1.754
Stroop^c^	−0.379	0.186	−0.351*	−2.034	−0.235	0.116	−0.346*	−2.017

*^a^Years of education.*

*^b^Matrix Reasoning Scaled Score.*

*^c^Stroop Color-Word Scaled Score. Standardized regression coefficients.*

*p < 0.05.

**Table 4:** Multiple linear regression analyses for cognitive-executive variables predicting SCL-90-R Global Severity Index in female premutation carriers.

	**Global Severity Index**			
**Predictor**	***B***	***SE B***	**β**	***t***
Age	0.015	0.167	0.015	0.093
YOE^*a*^	−0.076	0.157	−0.069	−0.485
MR SS^*b*^	−0.148	0.171	−0.863	0.849
Stroop^*c*^	−0.129	0.049	−0.328*	−2.475

*^a^Years of education.*

*^b^Matrix Reasoning Scales Score.*

*^c^Stroop Color-Word Scaled Score.*

Standardized regression coefficients. *p < 0.05.

## Discussion

The present study is the first to consider relationships between all three phenotypic domains– motor, cognitive-executive and psychiatric features, in a sample of non-FXTAS adult female PM carriers. These relationships specifically involved cognitive components representing aspects of executive functioning, including the Stroop Word-Color test of inhibitory control and several psychiatric symptom domains as measured by the SCL-90-R. Here we also provide evidence that this important component of executive functioning—the ability to inhibit cognitive interference—is significantly (negatively) correlated with the motor scales representing tremor/gait ataxia (ICARS) and parkinsonism (UPDRS) that are commonly used to evaluate motor dysfunction in PM carriers.

Our earlier study, based on data from the same sample of female PM carriers, found that a range of cognitive measures, especially those targeting executive functioning and psychomotor speed, were significantly and consistently correlated with these two motor scale scores (23). Here we expanded these results by including a measure of response inhibition using the Stroop Color and Word test, as well as a range of psychiatric features on the SCL-90-R. We showed significant correlations between both the Stroop and the Matrix Reasoning scores, and a range of psychiatric features on the SCL-90-R. Together these results provide convincing evidence that all three components—motor, cognitive-executive and affective (psychiatric) manifestations—are interrelated at a subtle yet detectable level in female PM carriers. It is of special interest that such a constellation of salient relationships between features of motor, cognitive and affective abnormalities is remininscent in a milder form to the constellation of cognitive impairments (particularly affecting executive functions), affective changes and motor dysfunction, specifically attributed to cerebellar damage (46).

Although we did not have direct evidence of specific cerebellar pathology in our female PM carriers (which would require MRI or brain biopsy), and none of the participants manifested obvious clinical symptoms of cerebellar ataxia, the results of the two motor scales applied in this study showed that nearly half of the carriers had at least one of these scores increased beyond two standard deviations, which clearly reflects some degree of cerebellar dysfunction (23). It should be acknowledged that the motor rating scales especially the ICARS, recognize the symptoms which occur, albeit in much more severe form, in clinically diagnosable FXTAS, and are associated with white matter lesions predominantly found in the middle cerebellar peduncles (15).

The current findings highlight the importance of impairment of the ability to ignore interference, as measured by the Stroop Color and Word test, in predicting certain psychiatric features on the SCL-90-R. These findings are consistent with a previous study in female PM carriers showing that psychological symptoms of anxiety and depression were highly elevated in those with poor response inhibition (7). Thus, the assessment of executive functions such as response inhibition could be useful in identifying risk factors for onset of significant psychiatric problems, especially anxiety and/or depression. Alternatively, these subtle executive function abnormalities may simply reflect the effects of psychological symptomatology on cognition. We may further speculate that subtle changes in the cerebellar peduncles in female PM carriers could give rise to mild cognitive regulatory impairments that interact with psychiatric symptomatology (perhaps via shared neuropathology) over time, and that in a subset of these carriers, may progress to a more severe clinical condition such as FXTAS. However, the possibility of the emergence and symptomatic expression of both cognitive inhibition impairments and psychiatric changes remains hypothetical, considering the cross-sectional nature of this study.

Given the multifaceted nature and fractionated components of executive functioning (47, 48), it is perhaps not surprising that some but not all cognitive-executive aspects might correlate with subtle motor features, as has been shown in our sample. The ability to suppress an unwanted response to avoid interference from prepotent stimuli, as assessed by the Stroop test, is commonly associated with recruitment of distinct regions in the ventromedial prefrontal cortex (49). However, we suggest that the cerebellum is likely to play an important role in inhibitory control by virtue of connectivity from medial prefrontal cortex through the pons and middle cerebellar peduncle via the cortico-ponto-cerebellar pathway (50). Although the presence of early cerebellar changes remains relatively unexplored in female PM carriers, there is evidence for subtle white matter alterations in the middle cerebellar peduncles in asymptomatic (non-FXTAS) male premutation carriers (51). In addition, the presence of abundant intranuclear inclusions has been reported throughout the brain in female PM carriers with and without FXTAS (52).

This study has a number of limitations. First, the reliance on self-reported psychological symptoms might be subject to biases and inaccuracy of participants' recall of their previous levels of psychological distress. Future studies applying semi-structured interviews will provide a more comprehensive evaluation; for example by using the Structured Clinical Interview for DSM-IV-TR (SCID) to confirm these relationships. Second, considering the variability of these measures, a larger sample size would provide more reliable results. Third, given the cross-sectional nature of this study, we could not assess the impact of executive function skills on psychiatric symptoms over time, and thus, were unable to determine whether the trajectories of any decline in motor function or cognition match those of any associated psychopathological impairments. Fourth, it should be acknowledged that the cohort of female PM carriers predominantly were mothers of children affected by FXS, hence our findings may not be generalizable to the broader population. Given the evidence of the effects of parenting stress of raising a child with a disability on increased risk for some psychiatric disorders (53, 54), future studies are needed in female PM carriers without children affected by FXS or other forms of disability. A final limitation is the lack of more sensitive and tract-based analysis of subclinical changes using advanced MRI techniques to examine relationships between microstructural alterations to the cerebellum and the executive-psychiatric phenotype in female PM carriers, but this preliminary observation may open the way for a future separate study.

In summary, our findings highlight the importance of response inhibition in the recognition of psychiatric symptoms and subtle tremor-ataxia motor features in female PM carriers subsymptomatic for FXTAS. We report interactive influences across the constellation of features reminiscent in a milder form in disorders associated with disruption to the cerebro-cerebellar circuits. Our findings of the involvement of all three interacting domains of PM carrier phenotype, together with motor involvement (tremor and cerebellar ataxia), suggest that these early changes may result from the subtle impact of PM alleles on early changes in the cerebro-cerebellar pathways. Advanced MRI techniques to assess middle cerebellar peduncles' white matter integrity may be able to strengthen these findings. Follow-up studies using longitudinal models is essential to verify the possibility that the subtle cognitive and psychiatric changes related to cerebellar involvement progress over time to overt and more severe neurological and executive-psychiatric dysfunction as seen in FXTAS.

## Data Availability Statement

The raw data supporting the conclusions of this article will be made available by the authors, without undue reservation.

## Ethics Statement

The studies involving human participants were reviewed and approved by La Trobe and Monash Universities Human Research Ethics Committees in Melbourne, Australia, and UC Davis Institutional Review Board, USA. The patients/participants provided their written informed consent to participate in this study.

## Author Contributions

DH: conception and wrote first draft of manuscript, conducting statistical analysis, interpretation of results, and final review and editing. DL: conception, organization and partial execution of research project, neurological assessments and motor scales scoring, review of statistical analysis, and co-writing the manuscript. PS: conduct and interpretation of neuropsychological and psychiatric pathology assessments and organization and partial execution of research project. FT: conduct and interpretation of genetic molecular assays and review and critique of manuscript. AA: contribution to cognitive testing and scoring, creating study database, and contribution to review and final editing of manuscript. ES: conception and partial execution of research project, neurological assessments and motor scales scoring, and neuropsychological assessments or supervision of assessments. All authors contributed to the article and approved the submitted version.

## Funding

This study was supported by the National Institutes of Child Health and Human Development Grant, US, No HD 36071, to DL and FT, and by National Health and Medical Research Australia project grant No CF06/0269 to ES, DL, and FT.

## Conflict of Interest

The authors declare that the research was conducted in the absence of any commercial or financial relationships that could be construed as a potential conflict of interest.

## Publisher's Note

All claims expressed in this article are solely those of the authors and do not necessarily represent those of their affiliated organizations, or those of the publisher, the editors and the reviewers. Any product that may be evaluated in this article, or claim that may be made by its manufacturer, is not guaranteed or endorsed by the publisher.
